# Construction of Ferric-Oxide-Doped Nickel–Iron Hydroxide Electrocatalysts by Magnetic-Field-Assisted Chemical Corrosion toward Boosted Oxygen Evolution Reaction

**DOI:** 10.3390/molecules29133127

**Published:** 2024-07-01

**Authors:** Mengdie Xu, Ling Lei, Huilin Hu, Yana Chen, Xuchao Yang, Kaige Yu, Bingying Cao, Xianzheng Zhang, Xueliang Jiang, Chu Yao, Huan Yang

**Affiliations:** Hubei Key Laboratory of Plasma Chemistry and Advanced Materials, School of Materials Science and Engineering, Key Laboratory of Green Chemical Engineering Process of Ministry of Education, Wuhan Institute of Technology, No. 206 Guanggu 1st Road, Wuhan 430205, China; 2105010723@stu.wit.edu.cn (M.X.); 13721592340@163.com (L.L.); 22105010012@stu.wit.edu.cn (H.H.); cyana2023@163.com (Y.C.); 13663482624@163.com (X.Y.); 13009665047@163.com (K.Y.); exatra15272116253@163.com (B.C.); zhangxianzheng1122@163.com (X.Z.); jiangxl@wit.edu.cn (X.J.)

**Keywords:** oxygen evolution reaction, chemical corrosion, magnetic field, transition-metal-based catalysts

## Abstract

Transition-metal-based oxygen evolution reaction (OER) catalysts have attracted widespread attention due to their inexpensive prices, unique layered structures, and rich active sites. Currently, designing low-cost, sustainable, and simple synthesis methods is essential for the application of transition-metal-based catalysts. Here, magnetic field (MF)-assisted chemical corrosion, as a novel technology, is adopted to construct superior OER electrocatalysts. The produced Ni(Fe)(OH)_2_-Fe_2_O_3_ electrode exhibits an overpotential of 272 mV at a current density of 100 mA cm^−2^, presenting a 64 mV reduction compared to the electrode without an MF. The experimental results indicate that an MF can induce the directional growth of Fe_2_O_3_ rods and reduce their accumulation. In addition, an external MF is beneficial for the lattice dislocation of the obtained catalysts, which can increase the surface free energy, thus reducing the activation energy and accelerating the electrochemical reaction kinetics. This work effectively combines a magnetic field with chemical corrosion and electrochemical energy, which offers a novel strategy for the large-scale development of environmentally friendly and superior electrocatalysts.

## 1. Introduction

Serious energy crises and environmental problems have hindered the sustainable development of the economy; thus, developing renewable and clean energy sources is vital to solve the above problems [1,2]. Hydrogen energy is widely used as a clean and renewable energy source [3]. Recently, environmentally friendly and sustainable methods of green hydrogen production, such as water splitting, have been developed [4]. However, as a four-electron transfer reaction, the oxygen evolution reaction (OER) (4OH^−^ → 2H_2_O + O_2_ + 4e^−^) is a key reaction during water splitting and generates a high overpotential. Therefore, developing highly efficient catalysts is essential to improve the slow kinetics of the OER [5,6,7]. Noble metals (such as Ru and Ir) demonstrate efficient OER activity; nevertheless, their low abundance, high cost, and instability limit their application [8]. NiFe compounds present the merits of low costs, adjustable components, and a controllable morphology; they are considered to be the most promising non-precious-metal-based OER electrocatalysts [9]. For instance, a two-stage electrodeposition technique was introduced to deposit individual Ru atoms on NiFe LDH. The obtained Ru_0.3_/NiFe showed an overpotential of 243 mV at 10 mA cm^−2^ and exhibited superior stability performance [10]. In addition, amorphous NiFe oxides synthesized by nanoreactors showed an overpotential of 228 mV at 10 mA cm^−2^ [11]. The superior OER performance of composite materials can be ascribed to the existence of strong interfacial interactions, which are beneficial for the transport of oxygen ions [12,13]. In addition, the strong electronic contact within the composite material can enhance the active sites, thus presenting high catalytic activity [14,15].

Recently, various synthesis methods, including high-temperature calcination, hydrothermal/solvothermal methods, and electrodeposition, have been widely adopted to construct OER catalysts. However, these preparation methods have the disadvantages of requiring harsh conditions, complex processes, and high energy consumption [16,17]. In general, the chemical corrosion of metals can spontaneously take place in the natural environment, which causes great damage during industrial production [18,19]. Typically, corrosion is a spontaneous redox reaction that occurs in different microscopic regions of a metal surface; this corrosion process can lead to the generation of metal oxide or hydroxide products [20]. Interestingly, these corrosion layers can be utilized as efficient OER catalysts; therefore, chemical corrosion, with its merits of a low cost, effective regulation, and large-scale production, can be adopted to synthesize superior OER electrocatalysts.

In addition, a magnetic field (MF) plays a critical role in synthesizing new materials and optimizing material properties. Generally, the high-intensity energy of MFs can change the microstructure of the material, such as the lattice arrangement, grain size, and orientation [21,22,23]. By adjusting the MF strength, the magnetic moment orientation and magnetic domain structure of the material can be regulated, thereby affecting the phase transition behavior of the material [24,25,26]. For example, a two-phase nickel–cobalt hydroxide nanosheet with a clear phase boundary was constructed by an MF-focused plasma jet [27]. Furthermore, there is a Lorentz force between the MF and the moving ions, which induces the ions to grow in a certain direction and form unique nanomaterials [28]. For instance, the MF-confined picosecond laser ablation of MOF was adopted to synthesize uniform ultra-small Co catalysts [29]. More importantly, an MF has the ability to greatly improve the substance transfer efficiency, thus affecting the formation of chemical corrosion products [30]. Therefore, MF-assisted chemical corrosion can serve as an important technology to construct advanced electrocatalysts.

In this work, the MF-assisted chemical corrosion strategy is introduced to construct efficient OER catalysts. The produced Ni(Fe)(OH)_2_-Fe_2_O_3_ electrode exhibits an overpotential of 285 mV at a current of 100 mA cm^−2^, marking a 45 mV reduction compared to the MF-free electrode. The experimental results indicate that an MF can induce the directional growth of Fe_2_O_3_ rods and reduce their accumulation. In addition, an external MF is beneficial for the lattice dislocation of the obtained catalysts, which can increase the surface free energy, thus reducing the activation energy and accelerating the electrochemical reaction kinetics. This work effectively combines a magnetic field with chemical corrosion and electrochemical energy, which offers a novel strategy for the large-scale development of environmentally friendly and excellent electrocatalysts.

## 2. Results and Discussion

**Figure 1:**
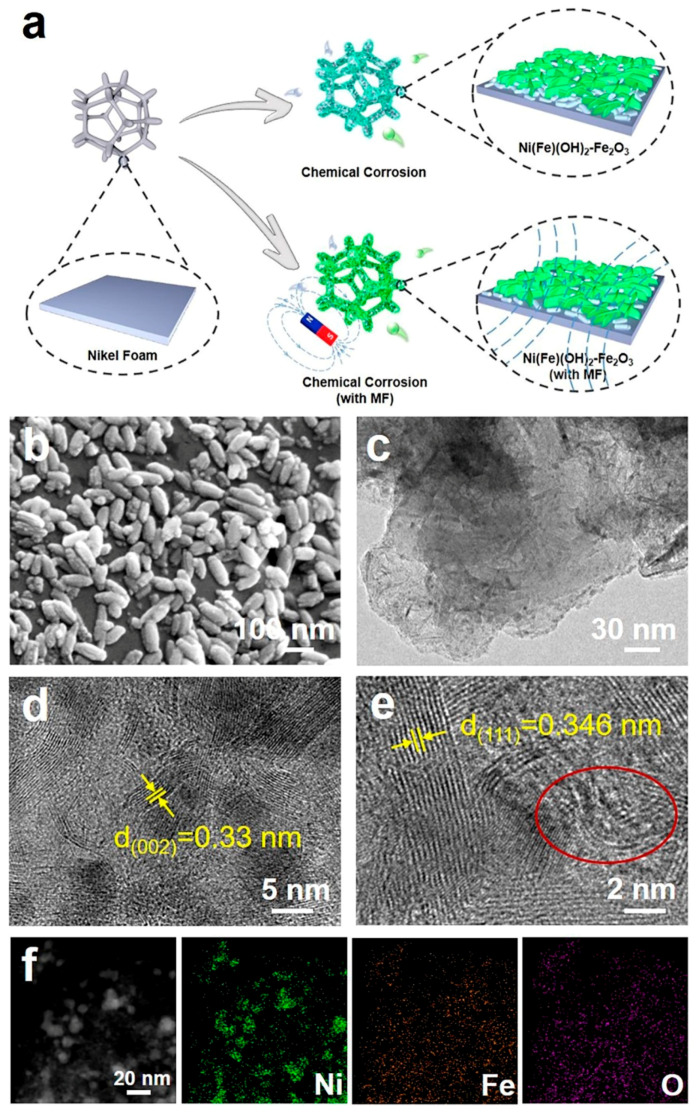
(**a**) Preparation process diagram; (**b**) SEM micrograph; (**c**) TEM micrograph; (**d**,**e**) HRTEM micrograph (yellow color: the lattice spacing, red circle: the lattice distortion region); (**f**) corresponding element mapping diagrams of electrocatalysts obtained under magnetic-field-assisted chemical etching.

When an external MF is applied to the system of NF immersed in an FeCl_3_ solution, chloride ions are hydrolyzed to produce HCl, which accelerates the corrosion of NF (Cl^−^ + H_2_O → HCl + OH^−^) [31]. In addition, OH^-^ from hydrolysis can react with the dissolved Ni^2+^ and Fe^3+^ in the solution to form NiFe layered double hydroxides (LDH) [32]. The high Fe^3+^ concentration can induce the resulted Fe(OH)_3_ to dehydrate to Fe_2_O_3_ (Fe(OH)_3_ → Fe_2_O_3_ + H_2_O) [33]. Under the presence of the MF, the magnetic induction line is perpendicular to the surface of the NF; therefore, the Fe_2_O_3_ nanorods can distribute on the surface of the NiFe LDH layer (Figure 1a). SEM and TEM images indicate that the corrosion layer presents some nanorods on the nanosheets (Figure 1b,c). After analyzing the images in high-resolution (HR)TEM, the lattice spacing of 0.33 nm is assigned to the (002) plane of the Fe_2_O_3_ nanorods, while the lattice spacing of 0.346 nm is assigned to the (111) plane of α-Ni(OH)_2_ (Figure 1d,e). Element mapping diagrams indicate that the Ni, Fe, and O elements are evenly dispersed on the corrosion product (Figure 1f). For the chemical corrosion environment without the external MF, there are denser Fe_2_O_3_ nanorods on α-Ni(OH)_2_ nanosheets (Figure S1), indicating that the MF can induce the directional growth of the Fe_2_O_3_ nanorods and reduce their accumulation. However, with the assistance of the MF, the lattice spacings of both Fe_2_O_3_ and α-Ni(OH)_2_ become larger, and there exists obvious lattice disorder (Figure 1e). This is ascribed to the directional moving ions in the solution, induced by the external MF. Furthermore, incorporating an MF may lead to a prolonged nucleation induction period, a widened substable zone, and an increased nucleation barrier. Under the joint influence of the nucleation kinetics and growth kinetics, an MF can have a significant effect on crystal growth and effectively control the crystal morphology [34,35]. Obviously, the atomic ratios of Fe to O in the electrodes constructed with an MF are significantly reduced, which further proves that the corroded electrodes constructed with an MF have lower Fe_2_O_3_ yields (Table S1). These results imply that the MF can modify the free energy of the synthesized substances and influence the nucleation selectivity accompanied by the nucleation rate of Fe_2_O_3_, effectively controlling the amount of Fe_2_O_3_ and avoiding its excessive stacking [36,37].

**Figure 2:**
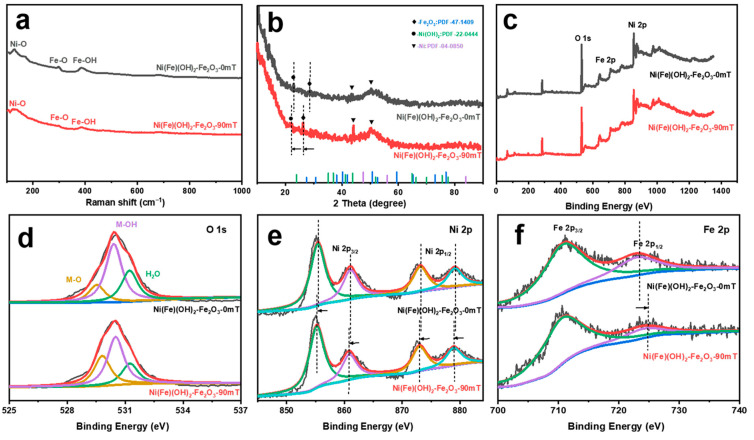
(**a**) Raman spectra; (**b**) XRD pattern; (**c**) XPS profile; (**d**) O 1s (yellow: M-O, purple: M-OH, green: H_2_O); (**e**) Ni 2p (purple: Ni 2p_3/2_, green: Ni 2p_1/2_); (**f**) Fe 2p (green: Fe 2p_3/2_, purple: Fe 2p_1/2_) of electrocatalysts obtained under different magnetic field strengths.

The samples have three characteristic peaks at 145, 300, and 400 cm^−1^ in the Raman spectrum, which correspond to Ni-O, Fe-O, and Fe-OH, respectively (Figure 2a) [38]. The peaks at 24° and 27° in the XRD correspond to Fe_2_O_3_ (JCPDS 47-1409) and α-Ni(OH)_2_ (JCPDS 22-0444) (Figure 2b). The peaks at 45° and 52° correspond to the substrate Ni (JCPDS 04-0850). Under the external MF, both the Fe_2_O_3_ peak and the α-Ni(OH)_2_ peak shift negatively, indicating that the crystal spacing increases. Moreover, the intensity of the diffraction peaks increases and they become sharper, which is ascribed to the enhanced crystallinity of the catalyst and the more regular internal atomic arrangement induced by the MF [36]. The coexistence of Ni, Fe, and O in the products is further verified by XPS (Figure 2c). The peaks at 529, 530, and 531 eV in the O 1s spectra correspond to M(Fe/Ni)-O, M-OH, and H_2_O, respectively (Figure 2d) [39]. The Ni 2p spectra present the characteristic peaks of Ni(II) (Figure 2e). The peaks at 855 and 861 eV are assigned to Ni 2p_3/2_, while the peaks at 873 and 879 eV are assigned to Ni 2p_1/2_ [40]. After adding the MF, these peaks shift negatively. The two characteristic peaks shown in the Fe 2p spectrum correspond to Fe(III) (Figure 2f). The peak at 711 eV is assigned to Fe 2p_3/2_, while the peak at 723 eV is assigned to Fe 2p_1/2_ [41]. With the addition of the MF, the Fe 2p_1/2_ peak is significantly shifted and has higher binding energy. The above results suggest that the introduction of the MF stretches the Fe-O bond in the structure, inducing the electrons to transfer from Ni to Fe through the O bridge across the Ni/Fe ions [42,43,44]. These results are consistent with the morphology characterization of the different corrosion electrodes.

**Figure 3:**
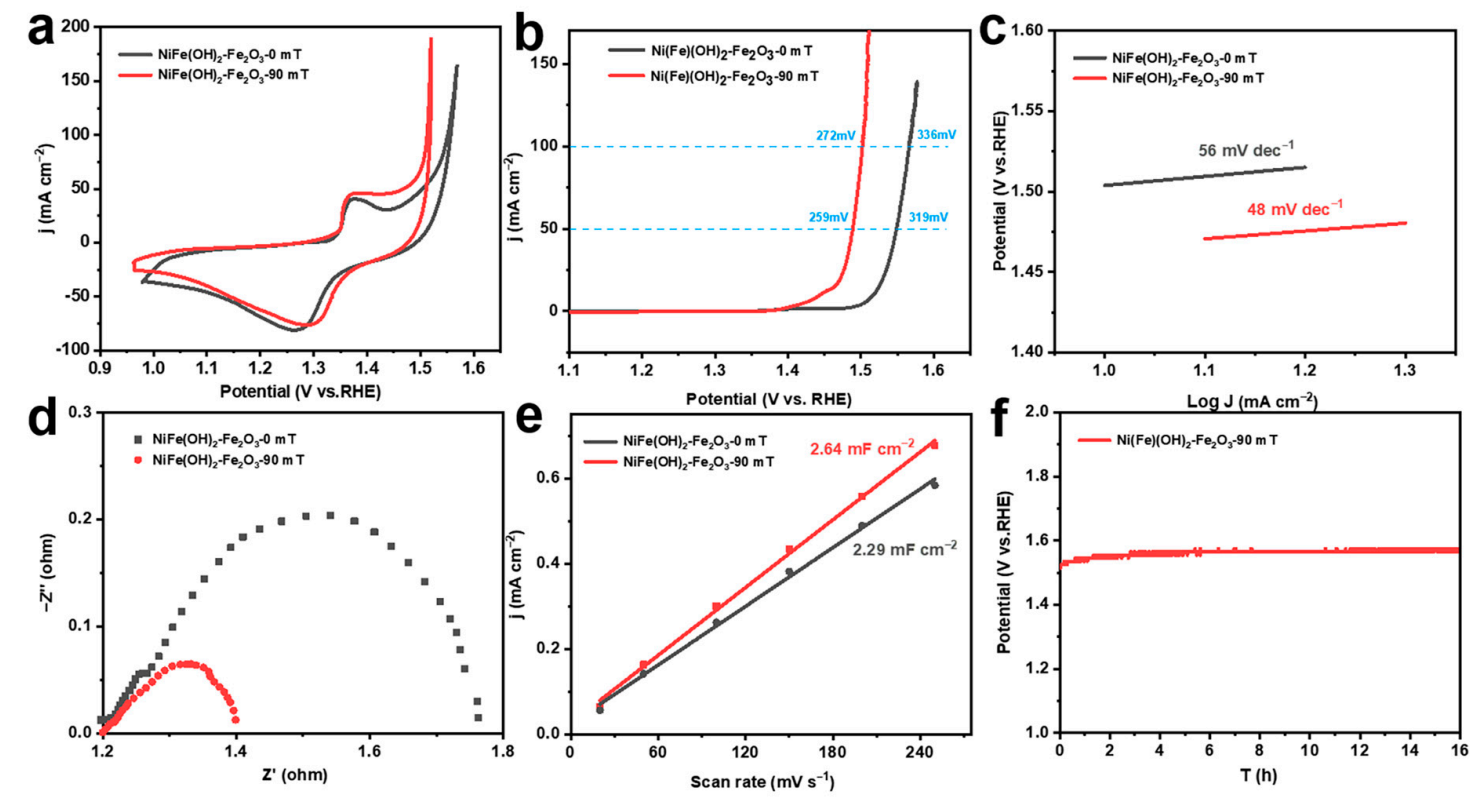
(**a**) CV curves; (**b**) LSV curves; (**c**) corresponding Tafel slope; (**d**) electrochemical impedance spectroscopy; (**e**) corresponding capacitance current scan rate curves; (**f**) chronopotentiogram at 100 mA cm^−2^ of electrocatalysts obtained under different magnetic field strengths.

The electrochemical measurements of different corrosion electrodes are conducted in 1.0 M KOH solution. Cyclic voltammetry (CV) is used for activation, and the oxidation peaks located at 1.2–1.5 V are assigned to the transformation of Ni^2+^ to Ni^3+^ (Figure 3a). The OER activity of the corrosion electrode is further evaluated by the LSV curve. The corrosion electrode constructed without an MF has an overpotential of 336 mV at 100 mA cm^−2^, while the corrosion electrode prepared with an MF shows a lower overpotential of 272 mV (Figure 3b). It can be seen that the corrosion electrode constructed with an MF has a Tafel slope of 48 mV dec^−1^, which is significantly lower compared to the MF-free electrode (56 mV dec^−1^) (Figure 3c) [45]. Comparing the electrochemical impedance spectra, the catalysts prepared with the external MF have lower charge transfer resistance (Figure 3d). In addition, the double-layer capacitances of the corroded electrodes increase, demonstrating that the OER catalysts constructed with the MF have a larger electrochemically active surface area (ECSA) (Figure 3e). The catalysts prepared with the MF still show better performance when normalized to the ECSA, implying that the Ni(Fe)(OH)_2_-Fe_2_O_3_ presents higher intrinsic OER activity (Figure S2). Furthermore, at a current density of 100 mA cm^−2^, the MF-assisted constructed electrode can remain stable for 16 h (Figure 3f). These results illustrate that the Ni(Fe)(OH)_2_-Fe_2_O_3_ constructed with an MF during the chemical corrosion process has outstanding OER performance and stability.

**Figure 4:**
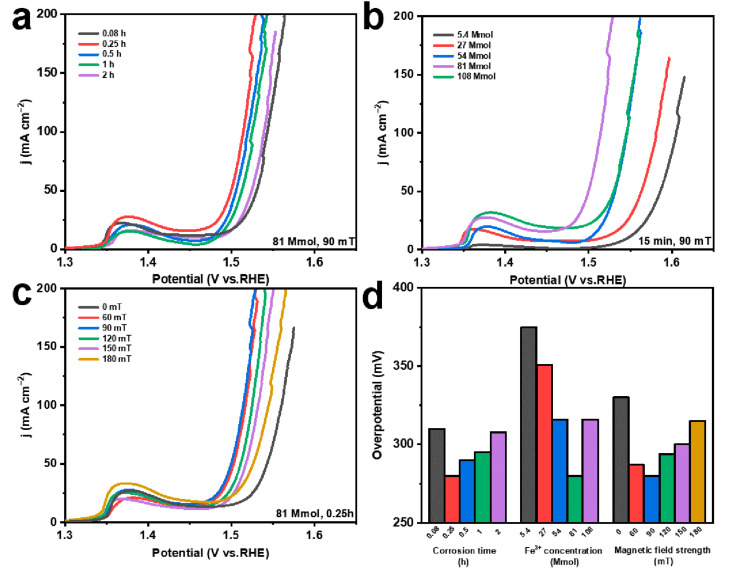
LSV curves of (**a**) electrodes prepared at different corrosion times; (**b**) electrodes prepared at different Fe^3+^ concentrations; (**c**) electrodes prepared under different magnetic field strengths; (**d**) overpotential at 100 mA cm^−2^ corresponding to electrodes obtained under different chemical corrosion conditions.

In order to illustrate the effects of various reaction environments and MF conditions on the performance of catalytic electrodes, different preparation conditions, including corrosion times (0.08 h, 0.25 h, 0.5 h, 1 h, and 2 h), FeCl_3_ solution concentrations (5.4 mmol, 27 mmol, 54 mmol, 81 mmol, and 108 mmol), and MF intensities (0 mT, 60 mT, 90 mT, 120 mT, 150 mT, and 180 mT), are considered. Under the external MF, the OER performance of the prepared electrodes is gradually improved with the increasing corrosion time from 0.08 to 0.25 h; then, the OER activity decreases from 0.25 h to 2 h, and the optimum OER performance is reached at 0.25 h (Figure 4a). The increased corrosion time induces more products, which can provide more exposed active sites (Figure 1b and Figure S3a). However, the continuously increasing corrosion time leads to the stacking coverage of the products, resulting in their gradual agglomeration into a dense lamellar structure (Figure S3b–d). Thus, the abundant active sites on the original NF surface are covered, reducing the OER activity of the catalyst [46]. Regarding the Fe^3+^ concentration in the corrosive environment, the OER performance gradually increases and then decreases, obtaining the optimum OER performance at 81 mmol (Figure 4b). This is mainly because more Fe^3+^ can form more Ni(Fe)(OH)_2_-Fe_2_O_3_ (Figure 1b and Figure S4a–c) [47,48,49,50]. Additionally, the generated heterojunction structures between Fe_2_O_3_ and Ni(Fe)(OH)_2_ can optimize the electronic structure of Ni in the electrode and further promote the OER performance [42]. Nevertheless, the higher Fe^3+^ can induce the formation of a disorganized lamellar structure, leading to fewer exposed active sites (Figure S4d) [46]. Concerning the external MF strength, the OER performance gradually increases with the increase in the MF strength from 0 mT to 90 mT, and the OER performance decreases when the MF strength increases to 180 mT (Figure 4c). The external MF can cause Fe_2_O_3_ to grow directionally and prevent stacking, thus increasing the exposed active sites of the electrode [51]. Moreover, the MF can promote the flow of ions in the solution, reduce the concentration of Fe^3+^ ions on the surface of the NF during chemical corrosion, and decrease the agglomeration of Fe_2_O_3_ (Figure 1b, Figures S1a and S5) [52]. However, when the external MF strength continues to increase, the ion movement in the solution is greatly promoted, which reduces the Fe^3+^ concentration and inhibits the Fe_2_O_3_ production on the NF surface. In addition, the overpotentials of different electrodes, at 100 mA cm^−2^, demonstrate more clearly the effect of corrosion conditions on the OER performance of the electrodes (Figure 4d).

**Figure 5:**
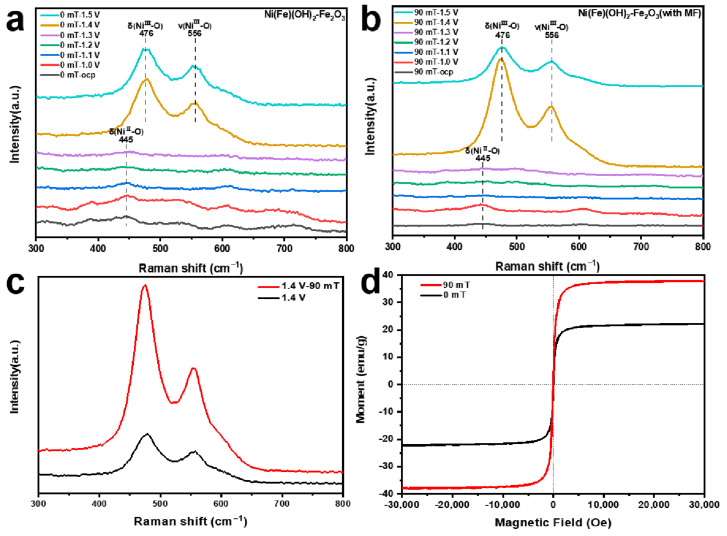
(**a**,**b**) In situ Raman spectra from open circuit voltage to 1.5 V vs. RHE; (**c**) in situ Raman spectra at 1.4 V vs. RHE; (**d**) magnetic hysteresis loops of electrocatalysts obtained under different magnetic field strengths.

To further assess the structural evolution of different samples during the OER process, in situ Raman spectroscopy from the open circuit voltage to 1.5 V vs. RHE is conducted (Figure 5a,b). When the actual potential exceeds 1.4 V vs. RHE, a pair of distinct Raman peaks appear at 476 and 556 cm^−1^. These peaks are ascribed to the bending and tensile vibrations of Ni(III)-O, suggesting that the Ni(Fe)(OH)_2_ transforms into Ni(Fe)OOH. In addition, at 1.4 V vs. RHE, the peak intensity of the electrode prepared with MF assistance is significantly higher than that prepared without an MF (Figure 5c). The applied MF can significantly accelerate the chemical corrosion process, which helps to control the generation and arrangement of the Fe_2_O_3_ [53]. These reactive substances can optimize the desorption of O intermediates in the OER process, which promotes the conversion of Ni(II) → Ni(III) [54]. As a result, the vibration of the chemical bonds inside the material becomes more intense, presenting a higher peak intensity in the Raman spectrum [55,56,57]. The magnetic properties of different catalytic electrodes are studied using a vibrating sample magnetometer. The electrode produced under the MF yields a higher saturation magnetization value (38 emu·g^−1^) (Figure 5d). The MF can induce directional alignment in the material [58]. For ferromagnetic materials, the spin magnetic moment makes an important contribution to the magnetic property, enabling the production of more free electrons [59,60]. Thus, the electrode has room-temperature ferromagnetism and there are more free electrons in the electrode. The external MF can promote the spin polarization of the electrons located in the outermost orbital and optimize the arrangement of the electrons, thus improving the OER performance [61,62,63].

## 3. Experimental Methods

### 3.1. Materials and Reagents

Ni foam (NF, 99.99%, Kunshan Long Sheng Bao Electronic Materials Co., Ltd., Kunshan, China) was used as the substrate. Other reagents and chemicals were of analytical reagent (AR) grade. Solutions were prepared with deionized water.

### 3.2. Corrosion Electrode Preparation

NF (1.0 cm × 5.0 cm) with a thickness of 1.5 mm was selected as the base material. The NF was placed in a mixture of anhydrous ethanol and acetone (volume ratio 1:1) for 30 min under ultrasonic treatment. Then, the pre-treated electrodes were immersed in an FeCl_3_ solution. Different corrosion times (0.08 h, 0.25 h, 0.5 h, 1 h, 2 h), Fe^3+^ concentrations (5.4 mmol, 27 mmol, 54 mmol, 81 mmol, 108 mmol), and magnetic field intensities (0 mT, 60 mT, 90 mT, 120 mT, 150 mT, 180 mT) were considered.

### 3.3. Physical Property Characterization

The morphology of various electrodes was observed by scanning electron microscopy (SEM, FEI NOVA NANO450, Hillsboro, OR, USA) and transmission electron microscopy (TEM, FEI-Talos F200X, Waltham, MA, USA). X-ray diffraction (XRD, Empyrean, Almelo, The Netherlands) was performed with Cu Ka radiation (λ = 1.5416 Å) and a scanning range of 10° to 90° at 10° min^−1^. Raman spectra (Horiba LabRAM HR Evolution, Paris, France) were collected at the wavelength of 532 cm^−1^. The base pressure of X-ray photoelectron spectroscopy (XPS, Kratos AXIS SUPRA, Kyoto, Japan) analyzed in the experimental chamber was below 10^−9^ bar, the spectra were measured with Al Ka (1486.6 eV) radiation, the overall energy resolution was 0.45 eV, and the binding energies were calibrated relative to the C 1s peak at 284.6 eV.

### 3.4. Electrochemical Performance Characterization

Electrochemical tests were carried out at the Chenhua Electrochemistry Workstation (CHI600E) with a three-electrode system. A synthetic corrosion electrode was used as the working electrode, a carbon rod was used as the opposite electrode, and a Hg/HgO electrode was used as the reference electrode. All tests were performed with 50 mL of KOH (1 mol L^−1^) as the electrolyte at ~25 °C and were repeated at least three times to ensure the reliability of the experimental results. Equation (1) was used to convert the potential data obtained from the electrochemical test into a relatively reversible hydrogen electrode (RHE) scale, and the oxygen evolution overpotential *η* = *E*_RHE_ − 1.23.

(1) $$E_{RHE}=E_{Hg/HgO}+0.0591\times PH+0.097$$

The electrochemical impedance spectroscopy (EIS) was measured at 1.54 V vs. RHE. The frequency range was 10^5^ kHz–0.01 Hz. The linear scan voltammetry (LSV) curves were measured at a scan rate of 5.0 mV s^−1^, starting from 1.0 V to 1.7 V vs. RHE. Cyclic voltammetry (CV) measurements were performed in the potential range of 1.07 to 1.18 V vs. RHE at different scan rates of 20, 50, 100, 150, 200, and 250 mV s^−1^, and 20 cycles were recorded. The OER stability of the electrode was tested at a constant current density of 100 mA cm^−2^.

## 4. Conclusions

In summary, an MF is introduced to further promote the chemical corrosion of NF, and the prepared electrocatalysts exhibit superior OER activity. The experimental results show that the Ni(Fe)(OH)_2_-Fe_2_O_3_ electrocatalyst exhibits an overpotential of 272 mV at a current density of 100 mA cm^−2^, marking a 64 mV reduction compared to the MF-free electrocatalyst (336 mV). These results indicate that MFs can induce the directional growth of Fe_2_O_3_ rods and reduce their accumulation, thus exposing more active sites. In addition, an external MF can induce the lattice dislocation of the heterojunction structure, which can increase the surface free energy, thus reducing the activation energy and accelerating the electrochemical reaction kinetics. This work demonstrates that an applied magnetic field can improve the composition and structure of catalysts during chemical corrosion, effectively combining the magnetic field with chemical corrosion and electrochemical energy, offering a novel strategy for the large-scale development of environmentally friendly and superior electrocatalysts.

## Data Availability

The most important data are included in this article, while others are included in the Supporting Information.

## Supplementary Materials

The following supporting information can be downloaded at: https://www.mdpi.com/article/10.3390/molecules29133127/s1, Figure S1: (a) SEM micrograph; (b) TEM micrograph; (c,d) HRTEM micrograph; (e) corresponding element mapping diagrams of the electrocatalysts obtained without MF; Figure S2: LSV curves of different samples after electrochemically active surface area normalization; Figure S3: SEM micrographs of electrodes prepared at different corrosion times of (a) 0.08 h; (b) 0.5 h; (c) 1 h; (d) 2 h; Figure S4: SEM micrographs of electrodes prepared at different solution concentrations of (a) 5.4 mmol; (b) 27 mmol; (c) 54 mmol; (d) 108 mmol; Figure S5: SEM micrographs of electrodes prepared at different magnetic field strengths of (a) 60 mT; (b) 120 mT; (c) 150 mT; (d) 180 mT; Table S1: The Content of Ni, Fe, and O elements corresponding to the element distribution of unactivated samples obtained by chemical corrosion under different magnetic field intensities.
