# Supplementary material for: Construction of Ferric-Oxide-Doped Nickel–Iron Hydroxide Electrocatalysts by Magnetic-Field-Assisted Chemical Corrosion toward Boosted Oxygen Evolution Reaction
Source: Molecules. 2024 Jul 1;29(13):3127. doi: 10.3390/molecules29133127 (PMC11242944; doi:10.3390/molecules29133127)
Supplement: Supplementary file 1 [file molecules-29-03127-s001.zip › molecules-3073051-supplementary.pdf]

Supporting Information

Construction of Ferric Oxide-doped Nickel-iron Hydroxides Electrocatalysts by Magnetic Field-assisted Chemical Corrosion toward Boosted Oxygen Evolution Reaction

*Mengdie Xu, Ling Lei, Huilin Hu, Yana Chen, Xuchao Yang, Kaige Yu, Bingying Cao, Xianzheng Zhang, Xueliang Jiang, Chu Yao * and Huan Yang **

Hubei Key Laboratory of Plasma Chemistry and Advanced Materials, School of Materials Science and Engineering, Key Laboratory of Green Chemical Engineering Process of Ministry of Education, Wuhan Institute of Technology, No. 206 Guanggu 1st Road, Wuhan 430205, China

* Corresponding author: C.Y. (email: chuyao@wit.edu.cn); H.Y. (email: yangh@wit.edu.cn)

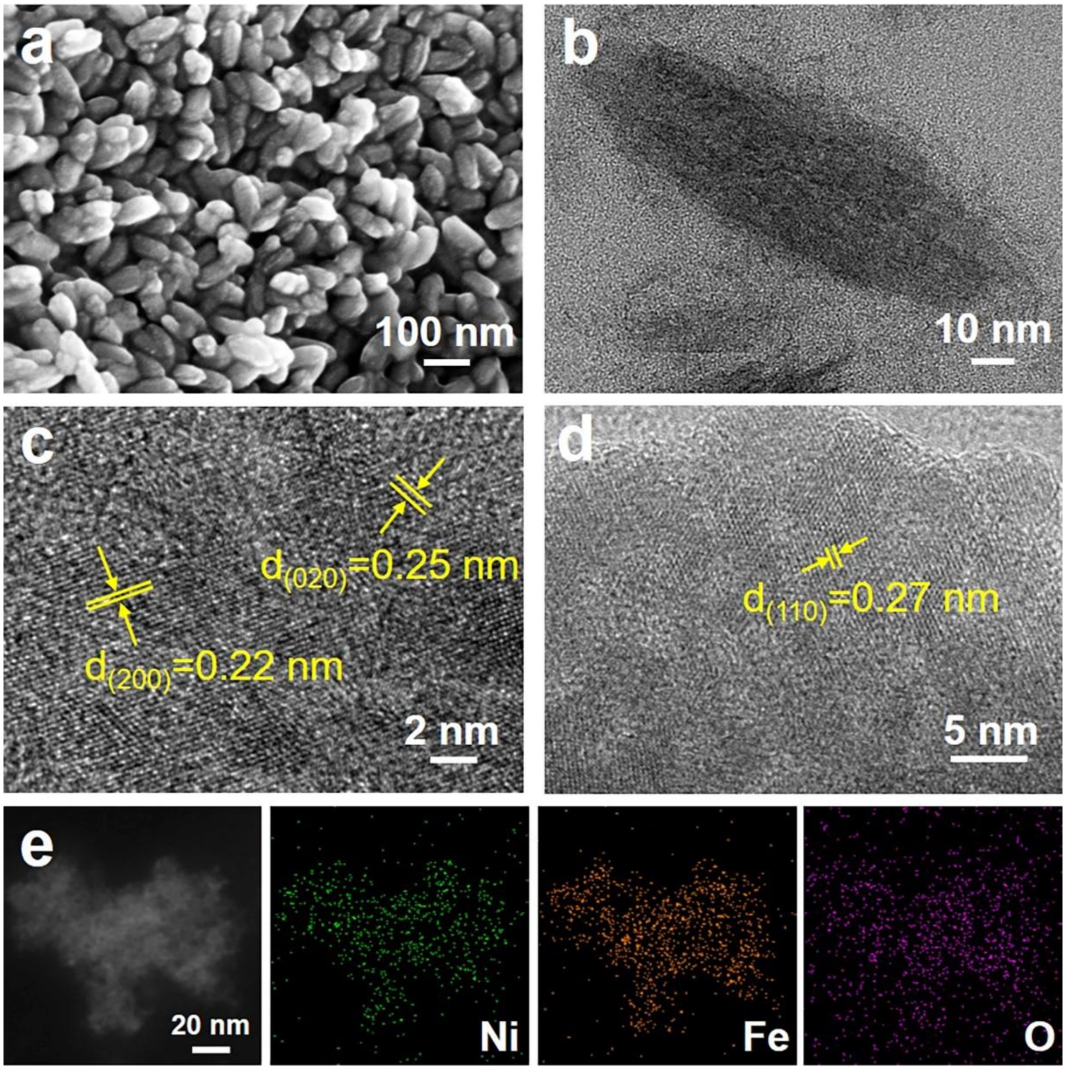

Figure S1. (b) SEM micrograph; (c) TEM micrograph; (d-e) HRTEM micrograph; (f) corresponding element mapping diagrams of the electrocatalysts obtained without MF.

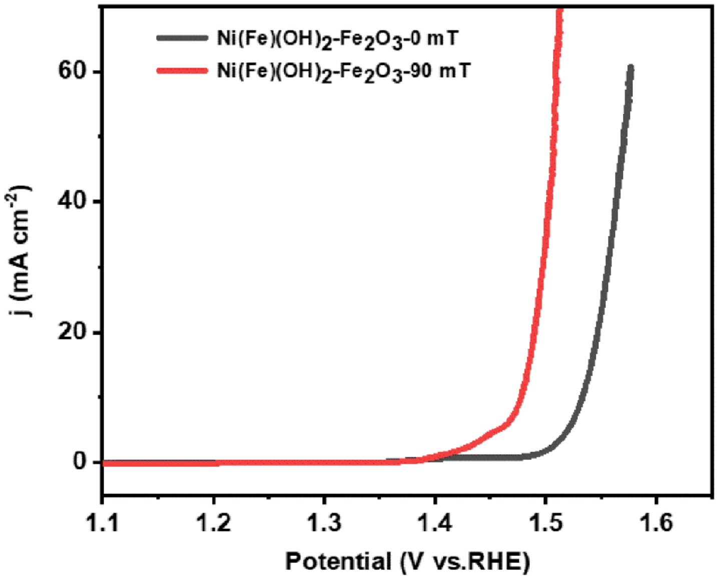

Figure S2. LSV curves of different samples after electrochemically active surface area normalization.

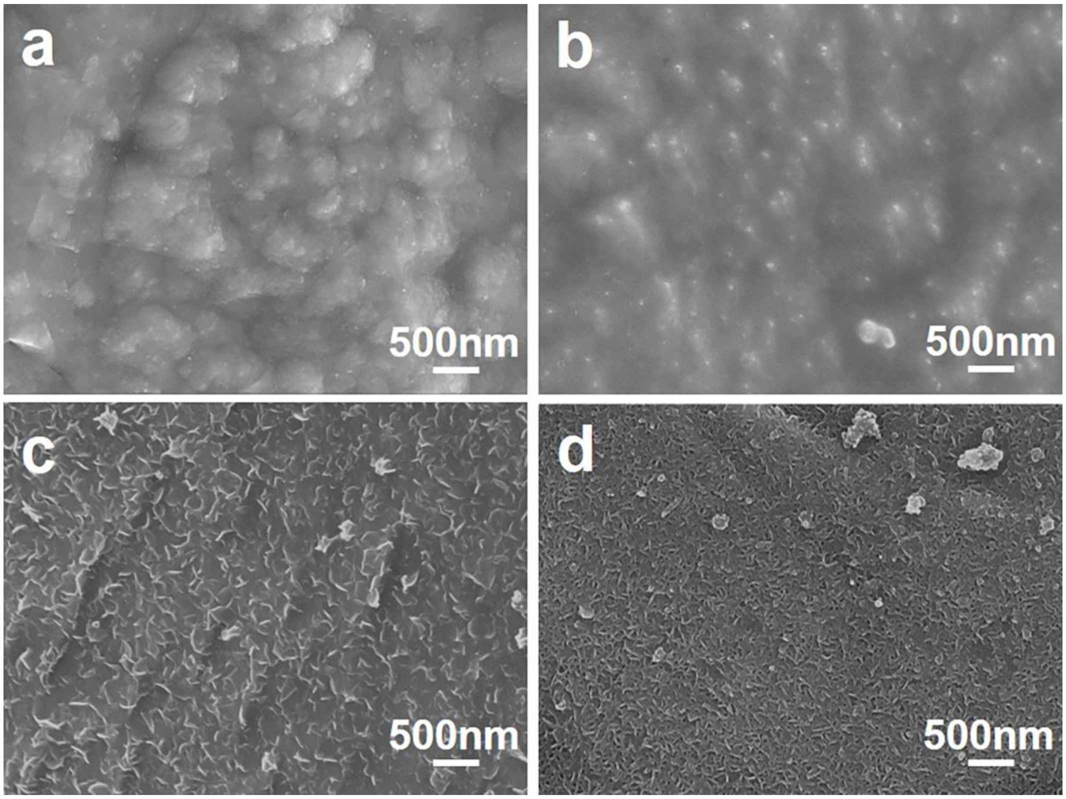

Figure S3. SEM micrographs of electrodes prepared at different corrosion times of (a) 0.08 h; (b) 0.5 h; (c) 1 h; (d) 2 h.

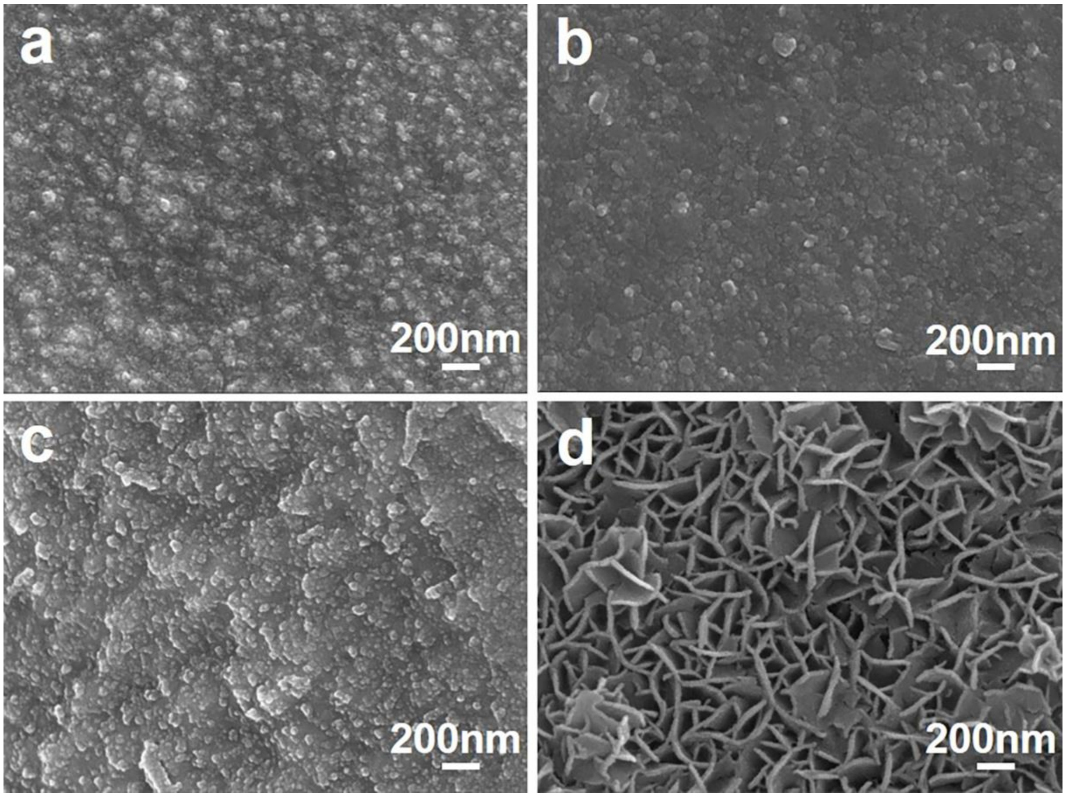

Figure S4. SEM micrographs of electrodes prepared at different solution concentrations of (a) 5.4 mmol; (b) 27 mmol; (c) 54 mmol; (d) 108 mmol.

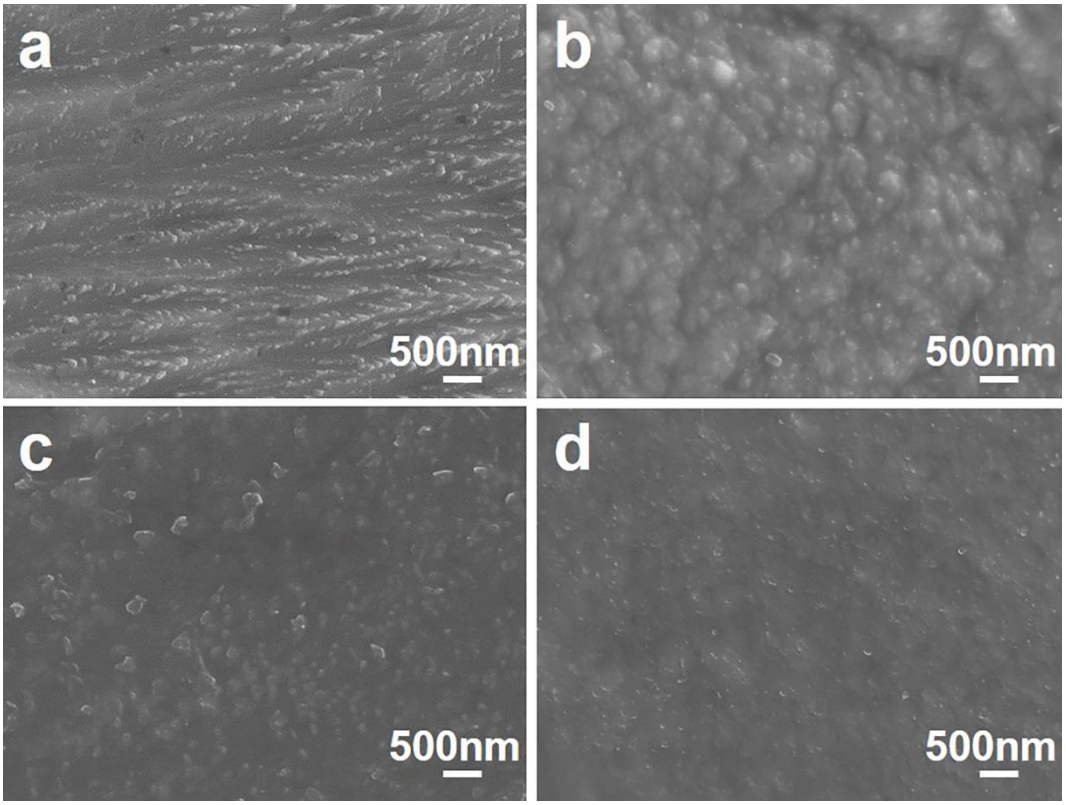

Figure S5. SEM micrographs of electrodes prepared at different magnetic field strengths of (a) 60 mT; (b) 120 mT; (c) 150 mT; (d) 180 mT.

Table S1. The Content of Ni, Fe, and O elements corresponding to the element distribution of unactivated samples obtained by chemical corrosion under different magnetic field intensities.

**Element**	**0 mT**		**90 mT**	
	Weight（%）	Atomic（%）	Weight（%）	Atomic（%）
**Ni**	16.06	7.71	37.94	19.63
**Fe**	44.19	22.29	27.64	15.03
**O**	39.75	70.00	34.42	65.34
